# The IL23-IL17 Immune Axis in the Treatment of Ulcerative Colitis: Successes, Defeats, and Ongoing Challenges

**DOI:** 10.3389/fimmu.2021.611256

**Published:** 2021-05-17

**Authors:** Daniele Noviello, Riccardo Mager, Giulia Roda, Riccardo G. Borroni, Gionata Fiorino, Stefania Vetrano

**Affiliations:** ^1^ Institute of Life Sciences, Scuola Superiore Sant’Anna, Pisa, Italy; ^2^ Inflammatory Bowel Disease (IBD) Center, Laboratory of Gastrointestinal Immunopathology, Humanitas Clinical and Research Center-IRCCS, Rozzano, Italy; ^3^ Inflammatory Bowel Disease (IBD) Center, Department of Gastroenterology, Humanitas Clinical and Research Center-IRCCS, Rozzano, Italy; ^4^ Department of Biomedical Sciences, Humanitas University, Pieve Emanuele, Italy; ^5^ Dermatology, Humanitas Clinical and Research Center-IRCCS, Rozzano, Italy

**Keywords:** ulcerative colitis, IBD, IL23, IL17, ustekinumab, mirikizumab, rinsankizumab

## Abstract

Ulcerative colitis (UC) is a chronic relapsing disorder of the colonic tract, characterized by a dysregulated innate and adaptive immune response to gut microbiota that contributes to the perpetuation of intestinal inflammatory processes. The Interleukin (IL) 23/IL17 axis has been reported to play a key role in UC pathogenesis promoting Th17 cells and cytokines-related immune response. Recently, the blockade of IL23/IL17 pathways has been raised enormous interest in the treatment o several chronic inflammatory disorders. In this review, we summarize the emerging results from clinical trials that evoked both promise and discouragement in IL23/IL17 axis in the treatment of UC. Targeting IL23 p40 through Ustekinumab results safe and effective to induce and maintain clinical remission, low inflammatory indexes, mucosal healing, and a better quality of life. Studies targeting IL23 p19 through Mirikizumab, Risankizumab, Brazikumab and Guselkumab are still ongoing. To date, no clinical studies targeting IL17 pathway are ongoing in UC. IL-17 targeting is thought to have a context-dependent biological effect, based on whether cytokine is selectively targeted or if its function is dampened by the upstream block of IL23.

## Introduction

Ulcerative colitis (UC) is a chronic relapsing inflammatory bowel disease (IBD), involving the rectum and a variable extent of the colon (1). It affects mainly young subjects (2^nd^-4^th^ decade of life, mainly), presenting with bloody diarrhoea (1, 2). Aminosalicylates and low-bioavailability corticosteroids are the main choice of treatment for mild to moderate disease, while systemic corticosteroids, immunosuppressants, monoclonal antibodies and small molecules are used in moderate to severe UC (3). Despite the fact that these agents have been proven to induce and maintain clinical and endoscopic remission (4, 5), the majority of patients lose response over time (5–8) and colectomy is needed in up to 15% of patients (9). Moreover, UC is a progressive and disabling disease with long term complications and often these agents fail to modify the course of the disease (10, 11).

Although still not completely clear, scientific evidences support a multifactorial pathogenesis characterized by a dysregulated immune response to gut microbiota which leads to progressive destructive damage and defective repair of the gastrointestinal tract (3). Immune cells, in fact, have primarily been explored as therapeutic targets to resolving their aberrant function in these patients. An excessive Th2 immune response with increased amounts of Interleukin (IL)13 and IL5 (12) is considered as a hallmark of UC. However, more recently, IL17-producing T cells, an independent lineage from Th1 or Th2 cells capable of promoting immune-mediated inflammatory responses in various immunological disorders (13–16), have been identified as new players in UC pathogenesis (16). The evidence that IL23 amplifies Th17 cell responses has opened new avenues to explore IL23/IL17 axis as promising therapeutic targets in IBD.

Several mouse models of colitis have shown an enhanced production of IL23 (17–20) and IL17 (21–23). Accordingly, all observational studies, despite the heterogeneity of the sample size (12 to 102 patients) and of the disease severity (active or remission), confirmed high levels of IL23 and IL17A in the serum of UC patients (24–30). Increased levels of IL23 correlated with disease severity in 40 UC patients (24). Nevertheless, data regarding both IL23 and IL17 expression in the inflamed mucosa remain controversial, probably due to the inhomogeneity in terms of sample size, clinical and endosopical activity and location of the biopsy (25, 29, 31). Moreover, the blockade of IL23 and to a certain extent IL17 effectively suppressed gut inflammation in various mouse models of colitis (18, 21, 32–37). These evidences supported the involvement of IL23 and IL17 in UC pathogenesis, therefore strategies aiming at their suppression have been posed as promising strategy for the treatment of UC.

In this review, we summarize briefly the IL23/IL17 pathway focusing on the emerging results from clinical trials that have evoked both promise and discouragement in IL23/IL17 axis in the treatment of UC.

## IL23/IL17 Pathways

**IL23** is a heterodimeric cytokine of the IL12 family composed of a specific p19 subunit and a shared p40 subunit (38, 39). Mainly produced by monocytes, macrophages, activated dendritic upon Toll-like receptor signaling (40), IL23 is capable to induce a strong proinflammatory effect through the activation of various target cells, beyond the aforementioned Th17 cells (41). IL23 binds to a heterodimeric receptor complex, IL23R, composed of the β1 subunit of IL12 (IL12Rβ1) and an IL23 specific subunit, (IL23Rα). Selected single nucleotide polymorphisms (SNPs) on IL23R gene have been associated to increased risk for the development of UC (42, 43), and to influence the phenotype of the disease (44). Interestingly, stratification by ethnicity revealed that some SNPs were high associated with UC in the Caucasian population, but not in Asians (45, 46), as if the mutation in IL23R increased predispositions for developing UC in certain geographic area.

Upon IL23 stimulation, the receptor activates the Jak-Stat signaling cascade promoting production of proinflammatory cytokines. Jak kinase 2 and tyrosine kinase 2 become activated and trigger the translocation of STAT3-STAT4 dimer to the nucleus, where in turn activates gene expression (39). IL23R is expressed on T cells, innate lymphoid cells, intraepithelial lymphocytes, natural killer cells, intestinal epithelial cells and granulocytes (41, 47). IL23-activated Th17 cells produce a variety of cytokines, including tumor necrosis factor-α (TNFα), INFγ, IL6, IL17A, IL17F, IL21 and IL22 (48, 49).

**IL17** is a cytokine family which comprises six proteins, IL17A to IL17F. **IL17A,** broadly distributed and produced by several cell types, is a potent pro-inflammatory cytokine that amplifies inflammatory response by sustaining the release of others inflammatory mediators such as TNF-α, IL-6 (16) and by inducing neutrophil-related genes like CXC-chemokine ligand 1 (CXCL1), CXCL2 and CXCL5 involved in the inflammatory processes (50). Activated neutrophils may prompt a positive feedback producing IL17A and IL22, as a result (51). Conversely, IL17A promotes antimicrobial or epithelial barrier genes like regenerating (REG) proteins, S100 proteins, lipocalin 2, lactoferrin, β-defensins, and claudin, zona occludens 1 (52, 53). **IL17F** shares almost half the structure of IL17A (54) and its effect on pro-inflammatory genes (CXCL1, IL6, CCL2, CCL7, and Matrix Metalloproteinases 13) with a less extent (23). **IL17B**, **IL17C** and **IL17D** are expressed mainly on epithelial cells and exert pro-inflammatory functions *in vitro*, but their exact biological roles have not yet been fully elucidated (55–57). Finally, **IL17E**, also known as IL25, is involved in Th2 cell responses against parasites (58).

## Interplay Between IL23/IL17 Axis and Gut Microbiota

The intestinal microbiota, in addition to having an enormous influence on nutrition, metabolism and physiology of the host, is also widely accepted as an immunomodulator of the development and maintenance of a healthy host immune system. The gut microbiota, in fact, has a pivotal role in the generation and functional training of innate and adaptive of immune cells, including Th17 cells, the most abundant CD4 T cells in mucosal tissues (59, 60). Accordigly, adult germ-free mice have fewer Th17 cells and smaller Peyer’s patches in their small intestine (61) confirming the crucial role of gut microbiota in the development of immune system. The transcription factor RORγt has been described to be important for Th17 differentiation by regulating the expression of Th17 genes and for the IL23/IL17 axis, key regulatory cells of the intestinal mucosal firewall, which provides a functional barrier of defense against microbial and dietary antigens by the presence of a mucus layer; the integrity of epithelial cells; and the release of antimirobial peptides and immunoglobulin A (62). Changes in the composition of microbial communities referred as dysbiosis can dictate intestinal immune response triggering immune diseases (63). Both commensal bacteria and pathogens can induce IL23 production by activated dendritic cells (64, 65). Recently, Martínez-López M et al. showed that the Mincle-Syk signaling axis is involved in the sensing of mucosal-associated bacteria through dendritic cells, which induce IL-6 and IL-23 first and then IL-17 and IL-22 production (66). Consequently, the absence of a functional Mincle-Syk axis is associated with impaired intestinal immune barrier function (66). Similarly, *segmented filamentous bacteria* (*SFBs*), *Cytophaga-Flavobacter-Bacteroidetes* are responsible for Th17 induction in the gut of adult mice (59, 67). Although the underlying mechanism is not well known, *SFB* overgrowth in mice with RORγt, IL-17 or IL-17R depletion has been found (68, 69). However, further studies are needed to address the active interplay between human IL23/IL17 and gut microbiota, for which limited studies are still available.

## Targeting IL23

### Anti-IL12/IL23 p40

Recently, **ustekinumab** (UST, Janssen-Cilag), a fully human IgG1κ monoclonal antibody against the shared p40 subunit of IL-12 and IL23 has been recently approved by EMA and FDA for treating of moderate to severe active UC who have had an inadequate response with, lost response to, or were intolerant to either conventional therapy or a biologic or have medical contraindications to such therapies. UST efficacy and safety has been investigated in a phase 3 trial (UNIFI) among 523 patients with moderate to severe active UC. Intravenous (IV) UST was more effective than placebo (15.6% vs 5.3%) for inducing clinical remission in patients at week 8. Subcutaneous (SC) UST q12w or q8w was more effective than placebo (38.4% or 43.8% vs 24%) for maintaining clinical remission in responders at induction at week 44. No significant differences were observed in patients with or without previous treatment failure with biologics (70). Among 116 delayed responders (pts achieving clinical response at week 16 continuing UST 90mg SC q8w) 74.1% were in clinical remission at week 44 and increased to 79.3% at week 92, among them 94.6% were corticosteroid free (71).

Very recently, further results from additional analysis on UNIFI data have showed its efficacy beyond clinical remission. Dose adjustment, based on the clinical judgement of disease activity, from UST q12w to q8w increased clinical remission rates (72). Reductions in stool frequency and rectal bleeding achieved after induction have been reported through 2 years of UST SC maintenance (73). Patients with mucosal healing, defined as Mayo endoscopy subscore ≤1 and histological improvement based on the Geboes score, after induction had significantly lower disease activity than those without at week 44, retained through week 92. A trend for lower inflammation measured by CRP and faecal calprotectin was also reported (74). Patients health-related quality of life (HRQoL), assessed using The Inflammatory Bowel Disease Questionnaire (IBDQ), and the The Short Form (36) Health Survey (SF-36), improved in most patients after UST induction therapy and was retained through week 92. 55.6% of patients were in IBDQ remission at week 92, 67.5% of them already in remission at maintenance baseline, and improvement in SF-36 (≥ 5 points) was achieved in half the patients (75). A pharmaeconomics analysis revealed that UST treatment in moderate to severe UC is cost effective *vs.* placebo over 1 year (76).

Real-world studies on UST efficacy are in progress. In the ENEIDA registry, among 47 patients previously exposed to biologics (>70% to >2), clinical response was achieved in 36% at week 8 (77). In the GETAID cohort, among 103 patients, most of them already exposed to anti-TNF and vedolizumab drugs, UST was effective in inducing steroid-free clinical remission and clinical remission in 35.0% and 39.8% respectively, at weeks 12–16. The endoscopic activity, assessed using the Ulcerative Colitis Endoscopic Index of Severity (UCEIS), showed a significant improvement from baseline, 3.8 ± 1.9 *vs.* 5.0 ± 1.2 (78). In two tertiary IBD centers in the US, among 66 patients almost all exposed to biologics or tofacitinib UST was effective in inducing clinical remission in 45% and 33% endoscopic and histologic remission at 1 year (79). Prior immunogenicity to anti-TNF did not confer a significantly risk of immunogenicity to UST in a cohort of 152 IBD patients, as the majority of real-worlds patients have likely failed anti-TNF biologics (80). Among 400 patients who received continuous UST in the induction, maintenance and LTE UNIFI trial, 22 (5.5%) patients developed antibodies to UST that were often transient and did not appear to affect efficacy or advers effects (81). Conversely, a smaller study found a strong association between antibodies to UST and clinical remission (82). Few case reports described the efficacious use of IV UST alone or in combination with cyclosporine as rescue therapy for acute severe UC (83, 84).

Phase 4 trials and observational studies are ongoing. BioIBD (NCT03885713) and i-BANK (NCT03809728) aim to identify predictive and prognostic biomarkers of natural history and response to biotherapies, including UST. VERDICT (NCT04259138) aims to define the optimal treatment target among corticosteroid-free symptomatic remission, or plus endoscopic remission, or plus histological remission. HARIR (NCT03006198) aims to explore the disease characteristics, treatment and outcomes in the emerging regions of North Africa, the Middle East, and Western Asia.

Because IL23 involvement and not IL12 seems pivotal in UC pathogenesis, a more selective generation of antibodies towards IL23 p19 is under investigation: mirikizumab, risankizumab, brazikumab and guselkumab.

### Anti-IL23 p19

**Mirikizumab** (LY3074828, Eli Lilly) is a humanized immunoglobulin G4–variant monoclonal antibody against the p19 subunit of IL23. In the phase 2 trial, mirikizumab did not achieve the primary endpoint, namely clinical remission at week 12, but it was more effective than placebo (59.7% *vs.* 20.6%) in inducing a clinical response with the 200-mg dose group showing the largest benefit. However, significant improvement in stool frequency and rectal bleeding were observed within week 2 and continued through week 52 (85). In the maintenance study, SC mirikizumab q4w in responders at induction increased clinical response and remission rates up to 80.9% and 46.8% at week 52, respectively (86, 87). Further analysis of the phase 2 trial data showed that endoscopic improvement and histologic remission were achieved respectively in up to 30.6% and 45.2% at the end of the induction phase and increased to 42.6% and 66.0% at week 52 (86, 87). Those results are consistent with significant improvements in patients HRQoL, assessed using the SF-36 v2, after 12 weeks of induction and sustained during the maintenance treatment (88). Absence of urgency is associated with improved clinical, endoscopic, histologic outcomes and better QoL assessed through the IBDQ (89, 90). In addition to the standard outcomes, additional exploratory biomarkers have been studied. IL17A and IL-22 plasma concentrations were reduced in clinical responders by 113.5%/57.4% at week 12 and further reductions were observed at week 52, leading to normal or near normal circulating levels (91). The genetic expression of biological pathways UC-specific and involved in resistance to anti-TNF showed a significant modulation in the inflamed tissue of UC treated with mirikuzumab for 12 weeks (92). Specifically, the different expression of genes involved in cell adhesion and leukocyte trafficking from UC inflamed tissue correlate better with histopatology than endoscopy and Mayo score (93).

Several phase 3 trials are ongoing. LUCENT 1 (NCT03518086) and LUCENT 2 (NCT03524092) are randomized, double-blind, placebo-controlled studies for induction and maintenance treatment, respectively, in patients with moderate to severe UC. LUCENT 3 (NCT03519945) is the long-term open-label extension program (NCT03519945). LUCENT-ACT (NCT04469062) is a randomized, double-blind, parallel-arm, placebo- and active- controlled treat-through study that aims to evaluate mirikizumab efficacy and safety compared to vedolizumab and placebo. SHINE 1 (NCT04004611) is a phase 2 Multicenter, Open-Label trial in Children and Teenagers (2 to 17 years).

**Risankizumab** (BI655066/ABBV066, AbbVie) is a humanized monoclonal antibody against the p19 subunit of IL23. To date, no results are available about its efficacy and safety in UC.

A Phase 2/3 randomized, double-blind, placebo-controlled trial for induction treatment (NCT03398148) and a Phase 3 randomized, double-blind, placebo-controlled trial for mainte- nance treatment (NCT03398135) are ongoing in moderate to severe UC. A phase 1 (NCT04254783) aims to evaluate the effect of IV infusions on pharmacokinetics of cytocrome p450 substrate.

**Brazikumab** (MEDI2070, AMG 139, AstraZeneca) is a human monoclonal antibody against the p19 subunit of IL23. To date, no results are available about its efficacy and safety in UC. An induction phase 2 multicenter, randomized, double-blind, double-dummy, placebo and active-controlled, parallel-group (NCT03616821, EXPEDITION) is ongoing in moderate to severe UC; vedolizumab is the active comparator. A phase 2 open-label extension study (NCT04277546) in patients of NCT03616821 trial who previously completed or discontinued brazikumab due to lack of efficacy after Week 10 is ongoing.

**Guselkumab** (CNTO 1959, Janssen-Cilag) is another human monoclonal antibody against the p19 subunit of IL23. To date, no results are available about its efficacy and safety in UC. A phase 2b/3, randomized, double-blind, placebo-controlled, parallel-group NCT04033445, QUASAR) is ongoing in patients with moderately to severely active UC. Combination therapy with guselkumab and golimumab is under investigation for the first time in a Phase 2a randomized, double-blind, active-controlled study (NCT03662542, VEGA in patients with moderate to severe UC).

To date, data on the safety profile of monoclonal antibodies targeting IL23 in UC comes only from UST e mirikizumab studies. UST safety profile has been relatively favorable in UNIFI. The incidence of serious adverse events and infections was similar to that with placebo, both in the induction and in the maintenance study. No malignancies, opportunistic infections or tuberculosis occurred (70). In the phase 2 trial, mirikizumab safety profile appeared consistent with other IL23-targeting biologics (86). The most frequent AEs were nasopharyngitis, worsening of UC, anemia, headache, nausea, cough, and worsening of gastroenteritis during induction; worsening of UC, nasopharyngitis, headache, upper respiratory tract infection, arthralgia, hypertension, and influenza during maintenance (86). UST exposure throughout pregnancy recorded no apparent safety signals (94). In particular, among 478 maternal pregnancies exposed to UST, 11 of them with UC, the prevalence of live births, spontaneous abortions and congenital anomalies were consistent with the general population and anti-TNF therapies. Real world data from the ENEIDA registry, the GETAID cohort and a US population were consistent with the known safety profile of UST (77–79). Three cases of leukocytoclastic vasculitis related to UST have been reported (79, 95, 96). To date, there are no data on safety for Risankizumab, Brazikumab and Guselkumab in UC.

A summary of all current clinical trials of anti-IL23 monoclonal antibodies in UC is shown in **Table 1**.

**Table 1:** Main characteristics of all ongoing clinical trials about monoclonal antibodies targenting IL23 p40 and p19 in UC.

Drug	Study Phase	Sample size (estimated primary completion date)	Primary endpoints	Active comparator	Reference
Ustekinumab	IV BioIBD	Recruiting (August 2021)	Identification of predictive Biomarkers for response to biologic therapies at induction	infliximab/adalimumab/golimumab/vedolizumab	NCT03885713
	IV i-BAnk	Recruiting (April 2021)	Identification of prognostic and predictive biomarkers of patients who have lost response to biotherapies	anti-TNF/ustekinumab/vedolizumab	NCT03809728
	IV VERDICT	Recruiting (November 2024)	Determination of the optimal treatment target among corticosteroid-free symptomatic remission, or plus endoscopic remission, or plus histological remission	/	NCT04259138
	Observational HARIR	140	Tracking biologics along the silk road: number of participants with clinical response, remission and quality of life.	remicade/simponi/stelara	NCT03006198
Mirikizumab	III LUCENT 1	Recruiting (September 2020)	Efficacy and safety of Mirikizumab to induce clinical remission at week 12	/	NCT03518086
	III LUCENT 2	Recruiting (March 2021)	Efficacy and safety of Mirikizumab to mantain clinical remission at week 40	/	NCT03524092
	III LUCENT 3	Recruiting (August 2023)	Evaluate the Long-Term Efficacy and Safety of Mirikizumab to mantain clinical remission at week 52	/	NCT03519945
	III LUCENT-ACT	Recruiting (March 2024)	Efficacy and safety of Mirikizumab to induce clinical remission compared to vedolizumab and placebo at week 12	vedolizumab	NCT04469062
	II	Recruiting (July 2022)	Evaluate Mirikizumab pharmacokinetics	/	NCT04004611
Risankizumab	II/III	Recruiting (March 2022)	Efficacy and safety of Risankizumab to induce clinical remission at week 12	/	NCT03398148
	III	Recruiting (December 2022)	Efficacy and safety of risankizumab to mantain clinical remission at week 52	/	NCT03398135
	I	Recruiting (July 2021)	Effect of Intravenous (IV) Infusions of Risankizumab on Pharmacokinetics of Cytochome P450 Substrates	/	NCT04254783
Bradizikumab	II	Recruiting (August 2020)	Evaluate the Efficacy and Safety of Bradizikumab to induce clinical remission compared to vedolizumab and placebo at week 10	vedolizumab	NCT03616821
	II open label	Enrolling by invitation	Safety of Bradizikumab	/	NCT04277546
Guselkumab	II/III QUASAR	Recruiting (June 2022)	Efficacy and safety of Guselkumab to induce clinical remission at week 12	/	NCT04033445
	II VEGA	Recruiting (November 2020)	Efficacy and safety of Guselkumab compared to vedolizumab and placebo to induce clinical remission at week 12	combination	NCT03662542

## Targeting IL17

To date, no clinical studies targeting IL17 pathway are ongoing in UC. However, preclinical models, human genetic evidences and clinical studies with anti IL17A/F in other immune-mediated inflammatory diseases (IMID) support a role of this pathway in the intestinal inflammation. In various mouse models of colitis, the blockade of IL17 pathways through monoclocal antibodies or genetic deletion led to contrasting results: from a protective to an irrelevant and even to an harmful effect have been described (21–23, 97, 98). Genetic studies on UC patients have showed that selected haplotype and polymorphisms in IL17 genes are associated with an increased susceptibility to UC (43, 99–102) and its severity (103, 104), although other studies did not detect these associations (105).

### Targeting IL17 in Crohn’s Disease

After the successful results of anti IL23 p40 and p19 monoclonal antibodies in reducing intestinal inflammation in Crohn’s Disease (CD) (70, 106) another form of IBD, targeting of the IL23/IL17 axis was thought to be a good strategy for both CD and UC. Further immunomodulation of the axis was attempted with the use of selective anti IL17A antibody and anti IL17R, secukinumab and brodalumab, respectively. Hueber et al. recruited 59 patients with moderate to severe CD and allocated them in a 2:1 ratio to receive secukinumab or placebo (107). The trial was prematurely stopped because an interim analysis found significantly higher rates of serious adverse events in the treatment group compared to placebo group (107). There is no trial evaluating the effect of Secukinumab in patients with UC.

Thus, future studies are evaluating therapeutic strategies combining IL17A and IL17F blockade.

### Targeting IL17 in the IMID

**Secukinumab** (Novartis), is an important pharmacological agent in the therapeutic armamentarium against psoriasis (PsO), psoriatic arthritis (PsA) and axial spondyloartropathies (axSpA). Schreiber et al. conducted a retrospective analysis of 21 trials evaluating the exposure adjusted incidence rates (EAIRs) of CD, UC and IBD unclassified (IBD-U) in 7355 patients with PsO, PsA or axSpA treated with secukinumab with a cumulative exposure of 16,226.9 person-years (108). In the PsO cohort (n=5,181) they reported 10 new-onset cases of UC and 4 exacerbations of UC (among 10 patients with a known history of UC) during the study treatment (EAIRs of 0.13 per 100 PY), in the PsA cohort (n=1,380) there were 2 new-onset UC cases and 1 exacerbation (among 2 patients with a known history of UC) (EAIRs of 0,08 per 100PY), in the Ankylosing Spondilytis (AS) cohort (n=794) they reported 3 new cases of UC and 1 exacerbations (among 3 patients with a known history of UC) (EAIRs of 0,2) [10]. In their analysis, the incidence rates of IBD were in the range of the background rates of the respective conditions, considering that patients with PsO, PsA and axial spondyloarthropathies have a 1 to 3 fold increased risk of developing IBD compared to the general population (109–111). Of notice, among 48 patients with an history of IBD at baseline, 11 had an exacerbation during the study period (22,9%) (108).

**Ixekizumab** (Eli Lilly and Co) is an anti-IL17A with proven efficacy in PsA, PsO and axSpA. Genovese et al. evaluated the result of 21 trials in PsA, PsO and axSpA patients exposed to Ixekizumab (112). Among the PsO patients (n=5898) there were 17 cases of UC (14 *de novo* and 3 exacerbations, IR 0.1 per 100 PY), in the PsA cohort (n=1401), they reported 1 case of new onset UC (IR <0.05 per 100 PY), in the axSpA population, 3 patients experienced *de novo* UC and 3 patients with a known history of UC reported an exacerbation (112). Unfortunately, the authors didn’t report the number of patients with a known IBD history at baseline.

**Bimekizumab** (UCB) is an anti IL17A and IL17F antibody currently in phase 3 clinical trials for AS, PsA and PsO [NCT03928743, NCT04109976, NCT03766685]. Dual neutralization of inteleukin-17A and inteleukin-17F with bimekizumab was tested in phase 2 studies in patients AS (113), PsA (114) and PsO (115). In the AS study, the authors report 4 cases of IBD (2 cases of UC) among 243 patients who received Bimekizumab (EAIRs for UC was 0.77 per 100 PY) (113). Notably, in the PsA and PsO study no cases of IBD were reported (in the PsA study 2 patients had an history of IBD (114, 115).

**Figure 1:**
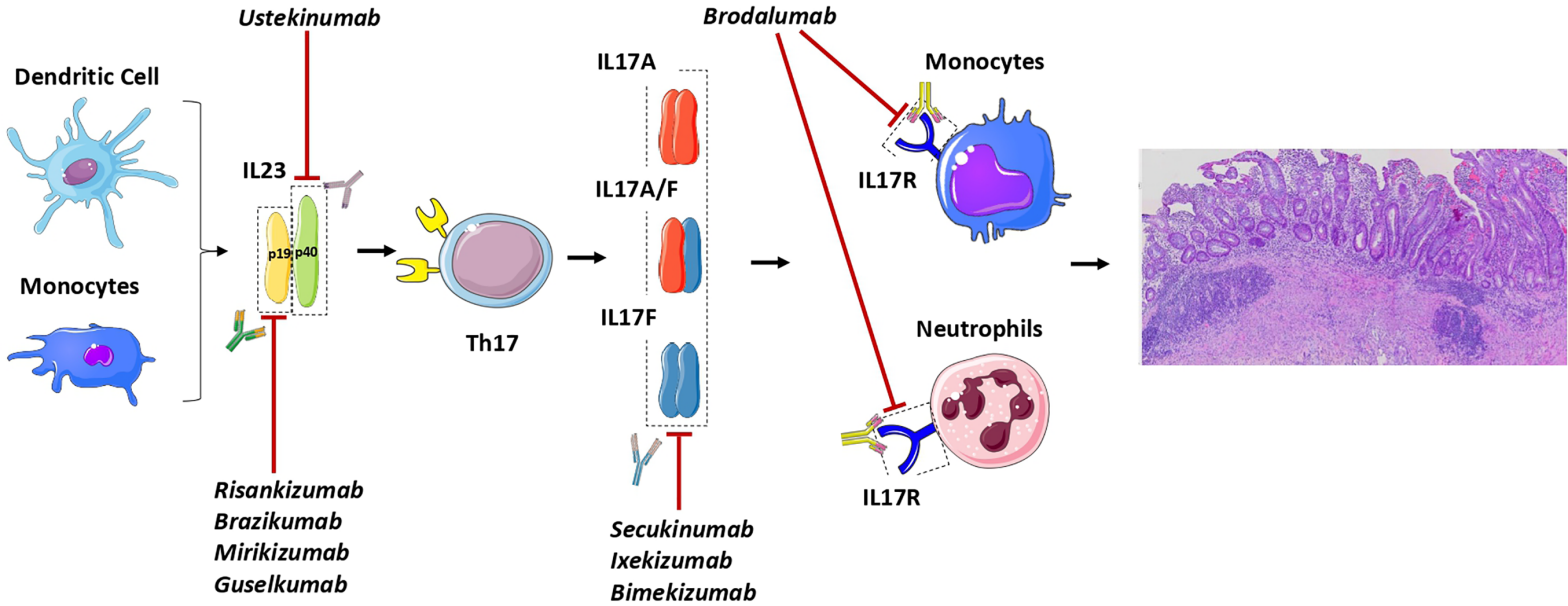
General outlook of the IL23/IL17 axis in Ulcerative Colitis and of the drugs active on this pathway. The monoclonal antibodies risankizumab, brazikumab, mirikizumab and guselkumab recognize specifically IL23 by binding to the p19 its subunit. On the other hand, ustekinumab bind to the p40 subunit which is shared by IL23 and also IL12. Downstream blockade of the pathway can be achieved with monoclonal antibodies targeting IL17 (secukinumab, ixekizumab and bimekizumab) and the IL17R (brodalumab) which is mainly present on neutrophils and monocytes.

A summary of all current drugs available towards IL23/IL17 axis is shown in **Figure 1**.

## Discussion

Since the discovery of the pivotal proinflammatory effect of TNF in the immune pathogenesis of UC, immunologists and clinicians have worked jointly to identity new targets as potential therapeutic strategies. The finding that the heterodimers alpha 4 and beta 7 integrins could mediate lymphocytes binding to the mucosal addressin cellular adhesion molecule-1 (MAdCAM-1) selectively in the gut has been another step forward. More recently, the increasing evidences of the IL23-IL17 axis involvement in UC pathogenesis have opened to several potential therapeutic options. Together, the pathways between the Janus kinase (JAK) family of tyrosine kinases and the signal transducer and activator of transcription (STAT) family of DNA-binding proteins have unlocked the potential to affect multiple pro-inflammatory cytokine-dependent pathways at once.

Several drugs with different mechanisms of action are currently available for patients with moderate-to-severe UC refractory to conventional therapies (116). However, a limited number of patients achieves and mantains remission on the long-term and a significative portion of patients develop complications such as proximal extension, strictures, pseudopolyposis, gut dysmotility, anorectal dysfunction, colectomy, hospitalization and colorectal cancer (3). Anti-TNFs (*e.g.* infliximab, adalimumab, golimumab) are all effective and safe in moderate-to-severe UC, both in patients naïve to biologics or previously exposed (4), and, in the case of infliximab, there is also evidence supporting their role as a rescue therapy in severe UC refractory to steroids (117, 118). Vedolizumab, a humanized immunoglobulin G1 monoclonal antibody to α4β7 integrin, is also effective in moderate-to-severe UC for those who are naïve or refractory to anti-TNFs (119). More recently, Janus Kinase inhibitors (anti-JAK) have been also approved for the same setting of patients (8).

Targeting IL23 have shown promising results from the clinical point of view. UST has been recently approved in moderate-to-severe disease, showing good efficacy profile both for induction and maintenance of clinical remission, mucosal healing, and histological response. On the other side, the safety profile of IL-23 shows no increased risks of side effects compared to placebo. UST is effective and safe both in naïve patients and in patients previously exposed to other monoclonal antibodies, positioning UST as first or second choice in the therapeutic algorithm. Preliminary data on other anti IL-23 agents also show promising results in terms of efficacy and safety. More data are needed on the long-term outcomes, such as prolonged remission, corticosteroid-free remission, hospitalization and colectomy rates, and safety, as well as direct comparison with other drugs approved for the same indication to understand the best positioning of those agent in the therapeutic algorithm. However, based on the paradigm shift towards precision medicine promoted by ECCO Scientific Workshop Steering Committee 2021 (120), patient specific characteristics should be considered more than drug characteristics. Given the beneficial effects in psoriasis and arthritis, UST treatment may be prioritized in IBD patients with extraintestinal manifestations. In addition, IL17A or IL-22 plasma concentrations at baseline could be eventually used to select patients as higher level of these citokynes were predictive for anti IL-23p19 success in CD patients (121).

To date, no clinical studies targeting IL17 pathway are ongoing in UC. However, the ineffective results of clinical trials on inhibition of IL17 in CD and the trigger effect on IBD onset or flare in patients treated for other IMID could be the clinical unmask of its context-dependent dual nature (16). IL17, independent from IL23, is involved in the local control of barrier integrity and defense against extracellular pathogens such as fungi and bacteria (122). In fact, genetic deficiency of IL17RA or IL17F is associated with chronic mucocutaneous candidiasis (122) and secukinumab -induced IL17F inhibition results in increased incidence of Candida spp. infection (107). IL17, dependent from IL23, exerts the known proinflammatory effect, successfully targeted in the other IMID. In addition, the remaining cytokines of the IL17 family may take part promoting inflammation in barrier organs or favoring repair of the gut mucosa after resolution of inflammation (50). An altered gut microbiota could be another possible explanation. In fact, Yeh et al. showed that treatment with secukinumab, but not UST, in psoriatic patients was associated at phylum level with increased *Proteobacteria* and decreased *Bacteroidetes* and *Firmicutes*, at family level with increased *Pseudomonadaceae* and *Enterobacteriaceae*, at order level with increased *Pseudomonadales* (123).

## Conclusions

Targeting IL23/IL17 is a promising new therapeutic approach for the treatment of UC. Currently, more than 150 clinical studies have been registered with the intention of discovering effective treatments, we will have to wait for the outcomes of these studies for better clarify the efficacy of this approach, and whether a profile of a patient’s gene variations can guide the selection of this treatment. Moreover, further studies are nedeed for the optimization of therapy, which demands a deeper understanding of disease mechanisms and drug modes of action that could support patient selection and treatment stratification.

## Author Contributions

DN and RM wrote the first draft and created table and figure. SV conceived the study and supervised the project. GR, RB, and GF critically reviewed the content of the paper. All authors contributed to the article and approved the submitted version.

## Conflict of Interest

RB is involved in educational programs promoted by Sanofi Genzyme, Janssen, AbbVie, Novartis, Almirall, and Pfizer. GF served as a consultant and a member of Advisory Boards for MSD, Takeda Pharmaceuticals, AbbVie, Pfizer, Celltrion, Amgen, Sandoz, Samsung, and Janssen Pharmaceuticals.

The remaining authors declare that the research was conducted in the absence of any commercial or financial relationships that could be construed as a potential conflict of interest.
